# Unveiling the role of adhesin proteins in controlling *Acinetobacter baumannii* infections: a systematic review

**DOI:** 10.1128/iai.00348-24

**Published:** 2025-01-08

**Authors:** Isabel Ladeira Pereira, Daiane Drawanz Hartwig

**Affiliations:** 1 Laboratory of Bacteriology and Bioassays, Federal University of Pelotas 37902 , Pelotas, Brazil; University of California Merced, Merced, California, USA

**Keywords:** bacterial adhesion, adhesion targets, immunotherapy, vaccine, biofilm, immunization

## Abstract

Combating multidrug-resistant *Acinetobacter baumannii* is considered a priority by the World Health Organization. Virulence mechanisms, such as biofilm formation, multidrug resistance, and high adherence to both biotic and abiotic surfaces, underscore the urgency of exploring approaches to control this pathogen. The search for new antibiotic compounds and alternative strategies like immunotherapies and vaccination offers potential solutions to address this pressing health concern. In this context, adhesins play a crucial role in the pathogenicity and virulence of *A. baumannii*, making them potential targets for therapeutic interventions. To address this, we conducted a systematic review of *A. baumannii* adhesin research from the last decade (2013–2023). We reviewed 24 papers: 6 utilizing reverse vaccinology bioinformatic tools to predict adhesin targets for vaccine construction, 17 employing DNA recombinant techniques for *in vivo* active and passive immunization or *in vitro* antibody-mediated therapy assays, and 1 paper exploring the impact of pyrogallol therapy on *A. baumannii* virulence mechanisms. Our review identified over 20 potential targets with significant findings. We screened and summarized these targets to aid in further exploration of therapies and prevention.

## ADHESINS IN *Acinetobacter baumannii*: KEY DRIVERS OF VIRULENCE

Efforts to combat the pathogenic species *Acinetobacter baumannii* have intensified in recent years, prompted by the World Health Organization’s designation of carbapenem-resistant *A. baumannii* strains as a priority for antibiotic development in 2024 (1). This Gram-negative bacterium represents a challenge to healthcare facilities, as it primarily affects individuals with compromised immune systems and possesses several virulence factors that contribute to its pathogenicity (2, 3). Biofilm formation, efflux mechanisms, high genetic plasticity to resist inhospitable environments, and the multidrug-resistant (MDR) phenotype are just some of the virulence factors that have earned *A. baumannii* the fame and nickname of “superbug” (4).

The emergence of *A. baumannii* urgently calls for efficient therapies or prevention. Besides new antibiotic treatments, there is a need for the development of alternative strategies, as current antibiotics become less effective year after year. Immunotherapies and vaccination emerge as viable alternatives, and with bioinformatics tools, it is possible to quickly scan the bacterial genome and select targets with vaccine candidate potential (5). Several aspects are important when considering a prophylactic vaccine. An ideal vaccine would be capable of protecting against all forms of infection. Additionally, it is crucial that the epitope is present on the bacterial cell surface and is expressed during human infection (6).

The ability to persist in the hospital environment is one of the main challenges in controlling *A. baumannii* infections (7). Since adhesion to host cells is the initial and pivotal step in host infection, numerous *A. baumannii* proteins involved in the adhesion of the pathogen to both biotic and abiotic surfaces have been studied to elucidate these virulence mechanisms (8). Once bacteria are already on the host surface, appendages, such as flagella and pili/fimbriae, can enhance irreversible attachment with adhesin proteins (9). Several specific interactions between bacteria and host cells are mediated by bacterial nanofibers, which are divided into two categories: fimbriae, assembled from subunits, and non-fimbrial fibers, which have monomeric or oligomeric structures (10). Fimbrial adhesins play roles in attaching to both biotic and abiotic surfaces, as well as in cell twitching motility, which directly enhances biofilm formation (11). On the other hand, non-fimbrial adhesins found in *A. baumannii* are also associated with biofilm formation and have a binding function to host extracellular matrix (ECM) components, such as fibronectin and collagen (10, 12). These polymeric fibers, besides their surface adherence function, also facilitate motility and plasmid transfer, thereby facilitating the spread of antibiotic-resistance genes (13).

Adhesin proteins are cell surface components closely linked to virulence. Without adhesin anchoring, bacteria would likely struggle to withstand shear forces such as the flow of fluids like blood and secretions (14, 15). In Gram-negative bacteria like *A. baumannii*, adhesins appear as fimbriae and pili proteins, while some enzymes and autotransporters also function as adhesins. These proteins differ in their architecture and receptor specificities, determining how they attach and bind to host cells (14, 16). The varied adhesin arsenal enables bacteria to establish their ecological niche during host infections through mechanisms such as adherence, invasion, survival, quorum sensing, biofilm formation, and serum resistance. Almost all regions of the human body are susceptible to inflammation triggered by adhesin-mediated immune activation, which is also associated with pathogen persistent as well. *A. baumannii* key adhesin ligands in host cells are the host ECM, fibronectin, integrins, selectins, and toll-like receptors (TLRs). These interactions activate innate immune responses, allowing healthy individuals to contain the bacteria through signaling receptors on epithelial and immune cells such as macrophages and neutrophils (14, 15, 17).

Identifying the main adhesins involved in the *A. baumannii* pathogenesis can provide insights for the development of effective preventive and therapeutic measures to control infections caused by this pathogen. In this study, we aim to review articles that investigate adhesin mechanisms in *A. baumannii*, focusing on controlling infections through vaccines and immunotherapies, as well as selecting targets for screening using bioinformatics tools, all while understanding the role of these proteins in the virulence and pathogenesis of the bacteria.

## STUDY INCLUSION CRITERIA AND REVIEW PROCESS

### Searching strategy

This systematic review was conducted following the Preferred Reporting Items for Systematic Reviews and Meta-Analysis guidelines. Scientific papers published between January 2013 and July 2024 were screened, focusing on articles from databases such as PubMed, Scopus, and ScienceDirect. The search employed specific keywords and Medical Subject Headings: “*Acinetobacter baumannii*” OR “*A. baumannii*” AND “immunization” OR “vaccine” OR “antibody” OR “immunotherapy” OR “immunoassay” AND “adhesin.” These terms were searched for in the title, abstract, and keywords.

### Inclusion criteria

The research papers included had to meet the following criteria: (i) scientific publications in the form of articles; (ii) original research papers, excluding review articles; (iii) studies published from January 2013 to July 2024; (iv) articles written in English; (v) *in vivo*, *in vitro*, and *in silico* studies that included adhesin proteins as a target to control infections caused by *A. baumannii*.

### Screening and selection

All the results found in the four different databases were collected in BibTeX format and added to a single library using JabRef Software. This tool performed an analysis of the library and removed duplicates. Subsequently, the reviewers screened the titles and abstracts to identify papers that matched the inclusion criteria. Studies that did not meet the inclusion criteria were excluded from further analysis.

### Data extraction

After selecting the publications, each full text was examined by the investigators. Collected data included the title, author names, year of publication, country of the research, adhesin targets, procedures, and key findings. Additionally, the reference sections were manually searched for potential additional papers.

## INSIGHTS FROM ADHESIN TARGETING IN *A. baumannii* CONTROL

### Selection of papers and data collection

**Fig 1:**
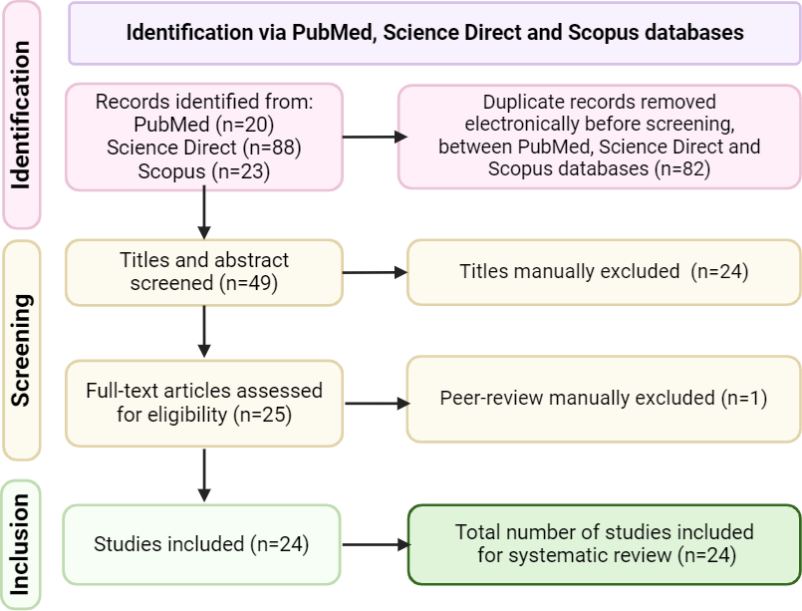
Workflow of this systematic review.

A thorough exploration across the four databases identified 131 studies. In this search, PubMed yielded 20 results, ScienceDirect presented 88 results, and Scopus unveiled 23 results. After assembling the library in Mendeley Reference Manager, 82 duplicates were removed. Furthermore, 24 studies were manually excluded through the screening of titles and abstracts, and one additional research study that lacked peer review was not considered. In the end, 24 studies were selected for full-text assessment and included in this systematic review. The detailed flowchart of the systematic review searching strategy is represented in Fig. 1.

*A. baumannii* represents a global threat to public health. In our search, we found research papers originating from nine different countries. Iran takes the lead, contributing nine studies (18–26). Following closely, India (27–30) and China (31–34) each have four papers included in this systematic review. Brazil contributes with three papers (35–37). Additionally, Finland (38), Taiwan (39), the United States of America (40), and Japan (41) each present one article, showcasing global collaboration in disseminating findings across various geographical regions.

To fully explore the findings of this systematic review, we categorized them into three groups: (i) articles using only bioinformatics tools to predict adhesins through *in silico* analyses; (ii) articles evaluating recombinantly produced adhesins; and (iii) studies using adhesin evaluation to control *A. baumannii*. We identified 15 studies aimed at exploring the potential immunoprotective effects of adhesin vaccines or immunotherapy, as well as investigating mechanisms of adhesin action within the immune system using recombinant DNA techniques. A summary overview of all included studies is available in Table 1.

**TABLE 1:** Summary of papers included in this systematic review^*g*^

Adhesin target	Year	Country	Reference
*In silico* approaches			
11 TonB-dependent receptors,**^*a*^** 2 fimbria or pilus-related proteins,**^*b*^** 8 porins,^*c*^ 7 efflux-related proteins,^*d*^ 3 lipoproteins,^*e*^ and 4 other putative extracellular proteins.^*f*^	2017	China	(32)
HP4 and HP8	2019	Iran	(21)
TAAs	2019	Iran	(19)
TAAs	2020	Iran	(20)
CarO FhuE, OmpH, FimF, and CsuB	2021	Iran	(24)
FilF	2022	India	(29)
Recombinant DNA technique			
FHA-like	2014	Iran	(18)
NucAb	2015	India	(27)
SurA1	2016	China	(31)
FilF	2016	India	(28)
Csu pili	2018	Finland	(38)
FilF and NucAb	2019	China	(33)
CsuA/B and FimA	2020	Iran	(22)
Csu pili	2022	Taiwan	(39)
CAM87009.1	2021	Brazil	(36)
Ata_263_	2021	Iran	(23)
InvL	2022	United States of America	(40)
CsuA/B and FimA	2022	Iran	(25)
Ataα	2022	China	(34)
AtaA	2023	Japan	(41)
OmpA	2024	Iran	(26)
CAM87009.1	2024	Brazil	(37)
FilF	2024	Brazil	(35)
Other study			
Bap, BfmR, OmpA, ClpB, CsuA/B, PgaA, and PgaC	2023	India	(30)

^*a*^ Hypothetical proteins (CAM86801.1, CAM88090.1, CAM86392.1, CAM86392.1, CAM86878.1, CAM85131.1, CAM87481.1, CAM86048.1, CAM86048.1, and CAM86923.1), BauA (putative ferric acinetobactin receptor), FhuE (Fe[III]-coprogen, Fe[III]-ferrioxamine B, and Fe[III]-rhodotrulic acid uptake), and Btub (putative TonB-dependent outer membrane receptor for vitamin B12/cobalamin transport).

^*b*^ CAM87009.1 and FilF (putative pilus assembly protein).

^*c*^ Hypothetical proteins (CAM87753.1, CAM88440.1, CAM86576.1, CAM85154.1, CAM85174.1, CAM85116.1, and CAM87023.1), OprB-like (putative glucose-sensitive porin).

^*d*^ Hypothetical proteins (CAM85825.1, CAM88576.1, CAM87663.1, CAM86485.1, and CAM87843.1), CzcC (cation efflux system protein), and ABC superfamily (toluene tolerance efflux transporter).

^*e*^ Hypothetical proteins (CAM86480.1, CAM87743.1, and CAM87612.1).

^*f*^ Hypothetical proteins (CAM85672.1, CAM88107.1, CAM85335.1, and CAM85336.1).

^*g*^ AtaA: *Acinetobacter* trimeric autotransporter adhesin; Ata_263_: *Acinetobacter* trimeric autotransporter adhesin short C-terminal extracelular region; Ataα: *Acinetobacter* trimeric autotransporter adhesin short C-terminal extracelular region; Bap: biofilm-associated protein; BfmR: biofilm-controlling response regulator protein; CarO: carbapenem resistance outer membrane protein; ClpB: probable ATP-dependent Clp protease; CsuA/B: chaperone-usher subunit A/B pilus protein; CsuB: chaperone-usher subunit B pilus protein; Csu pili: chaperone-usher subunit pili producing; FilF: pilus assembly protein; FimA: putative fimbrial protein precursor A; FimF: fimbrial protein F; FHA-like: filamentous hemagglutinin-like; FhuE: Fe(III)-rhodotrulic acid uptake; HP4: pilus assembly protein HP4; HP8: Bap-A-prefix domain-containing protein adhesin HP8; InvL: invasin-like adhesin; NucAb: *A. baumannii* nuclease; OmpA: outer membrane protein A; OmpH: outer membrane protein H; PgaA: biofilm PGA synthesis protein A; PgaC: biofilm PGA synthesis protein C; SurA1: surface antigen protein 1; TAAs: trimeric autotransporter adhesins.

### *In silico* methods to predict antigenic targets to *A. baumannii* vaccine constructions

Immunoinformatics approaches can enhance the success of vaccines. Traditional vaccine development typically involves lengthy periods due to extensive pre-clinical and clinical trial phases. However, *in silico* analyses can significantly shorten this process. This acceleration is facilitated by various computational methods that quickly identify immunogenic targets and ensure safety for humans or animals (42).

In our exploration, we uncovered six bioinformatics studies shedding light on the role of adhesins in combating *A. baumannii* infections. Among these, one study employed reverse vaccinology (RV) as a tool to predict 35 immunogenic adhesins from a pool of 33 *A. baumannii* genomes deposited on the National Center for Biotechnology Information(NCBI) (32). RV, which involves the analysis of genome protein-coding sequences to predict antigens for vaccine construction, has been applied to the development of vaccines against several pathogenic bacteria such as *Neisseria meningitidis* (43) and *Streptococcus pneumoniae* (44). Ni and colleagues screened 11 TonB-dependent receptor proteins, 2 fimbriae or pilus proteins, 7 efflux-related proteins, 8 porins, and other putative outer membrane and extracellular proteins. Notably, in curating these antigens, the researchers excluded proteins with low adhesin probability, demonstrating a strategic approach to antigen selection. They conducted an antibiotic resistance determinant analysis on the outer membrane and extracellular proteins, focusing especially on those with high adhesin probabilities, as they represent the most favorable vaccine candidates against antibiotic resistance. This study marks the first instance of using the reverse vaccinology strategy for systematic vaccine design against antibiotic resistance for any microbial pathogen (32).

Rahbar *et al.* conducted two studies to understand the role of trimeric autotransporter adhesins (TAAs) in *A. baumannii* infections (19, 20). The TAAs serve as notable virulence factors, facilitating bacterial adherence to surface components of host epithelial cells and thereby promoting biofilm formation (20). The analysis of gene acquisition in various *A. baumannii* genomes revealed that TAA genes were laterally acquired in environmental contexts, which incidentally provided benefits in host invasion. This explains why these genes are not present in many clinical strains (19). Subsequently, they determined that one component of the TAA family, the Ata_A_ protein, was conserved across several evaluated genomes in terms of protein domains. This determination was made through meticulous analysis of the domain architectures of TAAs and their conservation in sequences within the Moraxellaceae family. They discovered that Ata_A_ has multiple-binding sites, which assist the organism in attaching to different surfaces (20). In addition to structural analysis, these studies delved into the functional aspects, exploring the benefits of TAA gene acquisition in the development of biofilm communities and their adaptive strategies in various environments (19, 20).

In their bioinformatics analysis study included in this systematic review, Beiranvand *et al*. (24) aimed to identify antigen candidates for vaccine construction. Out of 15 antigens screened, six were selected based on their appropriate antigenicity, solubility, and immunogenicity. These proteins include the putative ferric siderophore receptor protein (Pfsr), lipopolysaccharide transport E (LptE), surface antigen (SurA), outer membrane protein H (OmpH), carbapenem resistance outer membrane protein (CarO), and the fimbrial protein F (FimF). Among them, OmpH, CarO, and FimF were identified as having the highest adhesion probability (cut-off: 0.51) in Vaxign analysis, while Pfsr, LptE, and FimF were found to be the most antigenic (24).

Another study screened 18 hypothetical proteins (HPs) from 30 *A. baumannii* MDR and extensively drug-resistant (XDR) genomes, identifying a total of 118 HP. Among these, 18 HPs possessed more than 200 amino acids and were selected for further analysis. This study represents the first report on the *in silico* investigation of common HPs in *A. baumannii*-resistant strains for drug and vaccine development. Seven proteins were chosen based on their theoretical potential involvement in the survival and pathogenesis of the bacteria. Among these, four were identified as potential vaccine candidates through antigenicity analysis. HP4 and HP8 appear to be associated with adhesion function, representing a pilus assembly protein and a BapA-prefix domain-containing protein, respectively (21).

Finally, the latest published paper, based solely on *in silico* tools, designed a multi-epitope-based subunit vaccine from the fimbriae pilus assembly protein, FimF. Using the native FimF sequence from the *A. baumannii* genome, they identified the best epitopes for vaccine construction. Subsequently, they conducted *in silico* cloning and immune simulation to evaluate vaccine efficacy. Additionally, they observed a stable interaction of the vaccine with human Toll-like receptor 4, an important factor in the host defense against *A. baumannii* (29). This collection of bioinformatics studies illuminates the crucial role of adhesins in *A. baumannii* infections, offering valuable insights for future research and therapeutic interventions.

### Investigating adhesin function in *A. baumannii* pathogenicity via recombinant DNA techniques

Among all the 21 studies under consideration, a predominant focus emerges on the application of recombinant DNA techniques**,** with 14 papers employing these approaches. These studies provide explanations for the various attributes that *A. baumannii* possesses and report, using recombinant DNA techniques, that these phenotypes are related to adhesive proteins (18, 22, 23, 25, 27, 28, 31, 33, 34, 36, 38–41). For example, Ishikawa *et al*. (41) investigated how the expression of the autotransporter adhesin (AtaA) protein varies across different growth phases of bacterial culture, providing insights into how cell growth and AtaA production interfere with biofilm formation and bacterial adhesion. This protein plays a significant role in mediating autoagglutination and adhesion to various surfaces. Initially, they observed that bacterial cells agglutinate via AtaA, forming small clusters that aggregate and develop into large clusters without a nucleus. However, these large clusters then precipitate and form biofilm on solid surfaces, demonstrating the crucial role of this adhesin protein in the primary steps of biofilm development (41).

Among the virulence factors of pathogenic bacteria, pili are an excellent choice of targets for vaccine development because they contain adhesins that mediate attachment to surfaces and contribute to biofilm formation, host colonization, and invasion (45). The *csu* gene cluster of *A. baumannii* type I pili has been extensively studied. The role of the Csu pili adhesin protein in biofilm formation on abiotic surfaces was also evaluated using recombinant DNA techniques by Pakharukova *et al*. (38). They expressed Csu pili and yellow fluorescent protein in *Escherichia coli* strains. This study elucidated that the unique binding mechanism of Csu pili enables tight attachment to structurally variable substrates, including polyethylene gloves. Targeting this binding mechanism can effectively block biofilm formation by this pathogen (38).

The utilization of knock-out strains could enhance studies on bacterial virulence by elucidating the specific roles of individual genes or proteins in pathogenic mechanisms. One study utilized the knock-out strain technique by generating a Csu pilus-producing operon knockout mutant *A. baumannii* strain and introducing the same gene into non-pilus-producing *E. coli*, comparing the effects on biofilm formation and adherence to pulmonary epithelial cells. Their findings unveiled a nuanced interplay between adhesin expression and bacterial behavior. The recombinant *E. coli* exhibited robust pilus production and a significant increase in biofilm formation and adherence capacity. However, while biofilm formation was meaningfully reduced, an unexpected increase in adherence to A549 cells in the knockout *A. baumannii* strain was demonstrated. This adds a layer of complexity to the adhesion dynamics (39).

The surface antigen protein 1 (SurA1) had its role in the pathogenicity of *A. baumannii* infections first reported through the *surA1* gene knock-out technique, revealing reduced biofilm formation ability and motility. Consequently, a correlation was established between the deficiency of the SurA1 protein and enhanced defense against bactericidal activity in human sera. Its virulence was also investigated by observing bacterial dissemination in *Galleria mellonella* hemolymph (31).

Jackson-Litteken *et al.* (40) researched *A. baumannii* uropathogenesis mechanisms, shedding light on a novel invasin-like adhesin, InvL. This adhesin was first identified as a substrate of the type II secretion system, a critical protein secretion system responsible for transporting proteins across the bacterial envelope and into the extracellular environment. Their study uncovers the significance of InvL in promoting bacterial adhesion and invasion in urinary tract epithelial cells *in vitro*. Additionally, when mice were implanted with a catheter followed by transurethral inoculation with an InvL knockout strain, researchers observed decreased binding to the catheter and reduced bladder colonization. These findings suggest that InvL could be a key virulence factor in *A. baumannii* uropathogenesis (40).

#### Adhesin-based subunit recombinant vaccines

Before vaccine candidates progress to clinical studies, it is essential to assess their *in vivo* reactions and immunogenicity in animal models (46). Vaccines contain immunogenic fragments of the pathogen to stimulate a protective immune response. Recombinant subunit vaccines offer advantages in terms of purity, safety, and stability, as well as highly precise recognition to induce an immune response (47).

Within the studies on active immunization utilizing recombinant adhesins as targets included in this systematic review, nine investigate the protective effect of antibodies against challenge with virulent strains of *A. baumannii* in mice (18, 22, 23, 25, 27, 28, 31, 33, 34, 37). Active immunization entails activating the immune system to generate immune defenses against specific pathogens. In this instance, recombinant proteins are employed to mimic native proteins and induce recognition of the bacterium by the immune system (48).

Remarkably, three of these vaccine investigations extend their scope to include passive immunization strategies (23, 25, 27). Passive immunization with pre-formed antibodies targeting outer membrane proteins has been considered a therapeutic approach for *A. baumannii*-resistant strains (49). This kind of immunization holds the potential to provide effective protection, either in combination with antibiotic therapy or independently. It has been widely used to delay bacteremia by reducing bacterial burdens. Although *A. baumannii* is responsible for nosocomial infections, developing a vaccine against this pathogen may not be feasible. However, active immunization can enhance the efficacy of antibiotic treatment in individuals at elevated risk of exposure to *A. baumannii* strains (48).

Darvish Alipour Astaneh *et al.* (2014) immunized mice with a recombinant filamentous hemagglutinin adhesin (FHA) carried by Freund’s adjuvant and found a 100% survival rate after 7 days of challenge with 10^10^ colony-forming units (CFUs) of the *A. baumannii* ATCC 19606 strain (18). FHAs are key factors in bacterial attachment and are also targets for vaccines that are currently licensed for use against other bacteria (50).

After the Csu knock-out strain demonstrated activity in attachment to human pulmonary cells and in biofilm formation (39). Ramezanalizadeh *et al*. (22) conducted an initial study to evaluate two recombinant constructs, CsuA/B and FimA, as targets to develop protective antibodies against a lethal sepsis dose of 10^6^ CFU *A. baumannii* ATCC 19606 mixed with 10% porcine mucin. Their findings revealed survival rates of 37% for mice immunized with rCsuA/B and 50% for those immunized with rFimA. Remarkably, mice immunized with a combination of rCsuA/B and rFimA exhibited a heightened survival rate of 62% (22). Subsequently, in another study by the same research group, they analyzed the combination of these targets, adding two recombinant proteins: one of the most important members of the iron-regulated outer membrane proteins family, the Baumannii acinetobactin utilization A (BauA) protein, and the putative heme receptor, HemTR. They found 87.5% survival of mice after 7 days of bacterial challenge with two clinical strains (ABI038 and ABI022) and bacterial burden clearance in the spleen, lungs, and liver samples of immunized mice. In passive immunization, the administration of immune serum led to 83.3% survival in mice challenged with the ABI038 strain and 16.66% survival in mice challenged with the ABI022 strain (25).

Ren *et al*. (33) undertook the construction of a vaccine formulation containing multi-epitopes derived from two adhesin proteins. They screened *A. baumannii* nuclease (NucAb) and pilus assembly protein (FilF) to select epitopes, and this formulation demonstrated a notable 88.9% survival rate in mice (33). Both antigens had been previously evaluated individually as targets for the immune response (27, 28). Singh *et al*. (28) reported a 50% protection rate against a lethal dose of 10^8^ CFU in a murine pneumonia model of *A. baumannii*, in mice immunized with recombinant FilF mixed with Freund’s adjuvant (28). In the same year, Garg *et al*. (27) assessed the immune protection of recombinant NucAb against the same lethal dose used by Singh *et al*. (28) but found only 20% survival in mice after intratracheal challenge. In passive immunization, mice treated with immune serum had an improved survival rate of 40% compared to the control group (27). Notably, both studies observed a significant decrease in serum pro-inflammatory cytokine levels, and histopathological analysis showed a reduction in the severity of the disease (27, 28). Both FilF and NucAb have been predicted to possess several attributes considered essential for vaccine candidates. Besides having a high probability of functioning as adhesins, these proteins exhibit non-homology to human proteins due to their localization in the outer membrane as fimbriae proteins (33). Following the same approach, in 2024, other fimbrial hypothetical adhesin was evaluated against *A. baumannii*, and 100% survival was observed in mice infected with *A. baumannii* MDR strain (37).

Most of the studies included in this review adopt the approach of mixing the recombinant antigen with Freund’s adjuvant. These substances, known as adjuvants, enhance the immunogenicity of antigens, thereby inducing stronger immune responses. Adjuvants also can increase the duration of an immune response to a foreign antigen (51, 52). Despite its ability to boost antibody production, Complete Freund’s adjuvant triggers multiple inflammatory effects, which is why its use in humans is prohibited (53). Only one study utilized an FDA-approved adjuvant: aluminum salts, which have been used in human vaccines for years. This study employed active immunization with a hypothetical fimbrial protein (CAM87009.1) and two adjuvants: an aluminum hydroxide composition and biogenic silver nanoparticles (bioAgNP), offering an experimental alternative adjuvant approach. While aluminum-based induce antibody-mediated immunity, they often lack robust cellular responses. Interestingly, mice immunized with aluminum as an adjuvant showed higher IgG levels than those receiving bioAgNP adjuvant. However, bioAgNP prompted higher IgM levels since day 0, indicating a less effective humoral response, as IgM does not provide long-term protection. Despite this, both groups achieved 100% survival after a bacterial challenge, suggesting probably a combination of humoral, cellular, or even innate responses, as IgM levels were already high on day 0, although this was not specifically tested (37).

Nanoadjuvants, such as bioAgNP, have been used to study T-cell responses. Polydopamine nanoparticles, a biopolymer derived from dopamine, have been described as a delivery system for intranasal adhesin Omp22 immunization, triggering strong Th1 and Th2 immune responses against XDR *A. baumannii* (54). Alternative strategies need to be explored to optimize antigen delivery to B and T cells and improve responses to combat *A. baumannii* infections. The pathogen-associated molecular pattern (PAMP) “Emulsan,” an extracellular acylated polysaccharide produced by *Acinetobacter calcoaceticus*, a close relative of *A. baumannii*, has been shown to activate macrophages in a dose-dependent manner, promoting Th1 response. Utilizing bacterial components and toxins can significantly enhance vaccine immunogenicity by recruiting innate immunity components such as antigen-presenting cells (55).

An alternative approach to enhancing the vaccine’s immunogenicity involves incorporating a built-in adjuvant into the designed vaccine, such as cholera toxin subunit B (CTB). CTB is a non-toxic subunit protein classified as a PAMP or TLR ligand. The CTB can bind to receptors in dendritic cells and B cell surfaces, which can offer advantages in vaccine delivery, endocytosis, and antigen presentation (52, 56). In a recent study by Sun *et al*. (34), the immunogenicity of a short C-terminal region of the *Acinetobacter* trimeric autotransporter adhesin (Ata) protein fused with CTB as an adjuvant was investigated. This innovative approach resulted in notable survival rates of 90% and 60% in mice challenged with a substantial bacterial load of *A. baumannii* ATCC 17978 and a clinical isolate XH733, respectively. Additionally, a significant reduction in bacterial loads was observed in the lungs, spleen, and bloodstream. These findings underscore the potential of CTB-Ataα to confer protective effects against *A. baumannii* infections, highlighting its promising aspects for clinical applications (34).

The first secretion system identified in *A. baumannii* was the Ata adhesin, which represents a domain associated with transport through membranes (57). Additionally, the passive administration of anti-rAta antibodies has already protected neutropenic mice from the *A. baumannii* challenge (58). The impact of this on Ata protein immunization was analyzed by Hatefi Oskuei *et al.* (2021), and the outcomes demonstrated notable variations in survival rates (23). Mice received rAta_263_ immunizations via subcutaneous, intraperitoneal (IP), and intranasal routes, with four doses each administered with Freund’s adjuvant. Additionally, two distinct routes were used in the sepsis model: a sublethal dose of *A. baumannii* ATCC 19606 mixed with 10% porcine mucin (vol/vol) was administered via intraperitoneal and subcutaneous routes. The study observed a notable survival rate of 66% in mice immunized via the subcutaneous and intraperitoneal routes. Remarkably, mice immunized via the intranasal route exhibited a 100% survival rate 6 months after the initial immunization. Passive immunization resulted in survival rates of 100% and 33.3% in mice that received sera from intraperitoneal and subcutaneous immunizations, respectively (23). Since mortality rates from *A. baumannii* infections are closely related to the respiratory tract, which serves as the natural and primary portal of entry (59), there is a growing need to analyze various vaccine administration routes. While the intranasal route enhances antibody protection against *A. baumannii* challenge (23), we observed a predominance of intraperitoneal vaccine administration, followed by subcutaneous, in the analyzed studies. It is known that the route of vaccine administration influences biodistribution, subsequent innate immune responses, and inflammation mechanisms, ultimately shaping the adaptive immune response (60).

We observe various bacterial challenge procedures, but most researchers opt for the IP route of administration for bacterial suspensions. Different doses, ranging from 10^4^ to 10^10^ CFUs, inoculated via the IP route have been observed. This variation could be attributed to the IP route allowing for higher volumes (61) and inducing a more severe form of infection, such as sepsis. However, the conditions encountered in human sepsis are not the same as those found in animal models, which are carefully selected and have similar genetic backgrounds, in contrast to the heterogeneous patient population with sepsis (62).

*A. baumannii* infections generally represent no threat to healthy individuals, as the pathogen activates the host’s innate immune responses (63). This activation leads to the production of proinflammatory cytokines and chemokines, as well as the local recruitment of macrophages and neutrophils. While this innate immunity can effectively manage and contain the infection, it may not be sufficient in immunocompromised individuals, allowing *A. baumannii* to evade host immune innate mechanisms (64, 65).

A significant challenge hindering drug development for *A. baumannii* infections is the lack of validated animal models for preclinical studies (66). Utilizing the appropriate animal model is crucial for understanding the progression of *A. baumannii* and for identifying prevention and treatment strategies (67). Determining the ideal animal model for *A. baumannii* infection can be challenging because the majority of *A. baumannii* clinical isolates display virulence primarily in immunocompromised individuals (68). Key factors influencing the efficacy of *A. baumannii* animal models include the virulence of the bacterial strain, the route of infection, and the administration of immunosuppressive drugs or virulence-enhancing agents (62). Host neutrophils play a significant role in resistance to *A. baumannii* infections, which has led to the use of neutropenic models achieved through neutrophil depletion. Cyclophosphamide and monoclonal antibodies have been employed to induce neutropenia for studying the pathogenesis of *A. baumannii* in host cells (68, 69).

Using a different approach, three articles included in this review employed porcine mucin to increase virulence, which allows for reducing bacterial concentrations in challenge assays (22, 23, 25). Most *A. baumannii* strains exhibit low virulence in conventional mice, with exceptions found in known hypervirulent strains. As a result, researchers often utilize immunocompromised mice or inoculate porcine mucin mixtures, which provide a practical and cost-effective approach to studying virulence and pathogenesis (70)

The use of porcine mucin mimics the natural environment encountered by the bacteria in the host. Intraperitoneal injections of porcine mucin induced a significant reduction in peritoneal macrophages and lymphocytes, accompanied by a significant recruitment of neutrophils. This distinct approach could enhance the virulence of different strains of *A. baumannii* in BALB/C and C57BL/6 mice (70). The expression of immune and antioxidant genes in macrophages treated with porcine mucin showed downregulation of anti- and pro-inflammatory chemokines and antioxidant enzymes, potentially leading to a reduction in immunological parameters (71).

By enhancing the pathogenicity of *A. baumannii* in mouse models, they become better representatives for studying human infections, especially in the sepsis model.

All the information regarding active and passive immunization trials included in this review is summarized in Table 2.

**TABLE 2:** Complete screening of adhesin immunization trials^*c*^

Adhesin target	Type of immunization	Number of doses/antigen concentration per dose	Route	Adjuvant	Challenge dose and strain/route	Survival^*a*^	Reference
FHA-like	Active	4/20 µg	IP	Freund’s adjuvant	10^10^ CFU ATCC 19606/IP	100%	(18)
NucAb	Active	3/25 µg	IP	Freund’s adjuvant	10^8^ CFU ATCC 19606/IT	20%	(27)
	Passive	100 µL of immune serum 4 hours before bacterial challenge	IV	–^d^		40%	
FilF	Active	3/20 µg	IP	Freund’s adjuvant	10^8^ CFU ATCC 19606/IT	50%	(28)
FilF and NucAb	Active	3/30 µg	IP	Freund’s adjuvant	2 × 10^8^ CFU ATCC 19606/IP	88.9%	(33)
CsuA/B, FimA, and Csu-Fim	Active	3/10 µg of each protein and both combined (Csu-Fim)	SC	Freund’s adjuvant	1.2 × 10^6^ CFU ATCC 19606 with 10% porcine mucin/IP	37%: CsuA/B 50%: FimA 62%: Csu-Fim	(22)
Ata_263_	Active	4/30 µg	SC, IP, and IN	Freund’s adjuvant	1.2 × 10^6^ CFU ATCC 19606 with 10% porcine mucin/IP	66%: SC and IP 100%: IN	(23)
	Passive	100 µL of immune serum 3 hours before bacterial challenge	IV	–		100%	
CsuA/B, FimA, HemTR^*b*^, and BauA^*b*^	Active	3/10 µg of each protein	SC	Freund’s adjuvant	10^4^–10^6^ CFU of different Ab clinical isolates with 10% porcine mucin/IP	87.50%	(25)
	Passive	100 µL of immune serum 3 hours before bacterial challenge	IV	–		83.3%	
Ataα	Active	3/40 µg	IP and SC	CTB adjuvant	2 × 10^7^ CFU ATCC 17978/IP	90%: IP	(34)
						60%: SC	
CAM87009.1	Active	3/25 µg	IM	Alhydrogel and bioAgNP	10^4^ CFU of Ab clinical isolate/IP	100%	(37)

^*a*^ Survival rate in immunized groups.

^*b*^ Non-adhesin proteins used in vaccine formulation.

^*c*^ Ab = *A. baumannii*; bioAgNP = biogenic silver nanoparticle; IN = intranasal route; IM: intramuscular route; IP = intraperitoneal route; IV = intravenous route; IT = intratracheal route; SC = subcutaneous route.

^d^ The en dash used indicates that "no adjuvant was used" in those instances.

#### Antibodies treatment overcomes pathogens in immunoassay

Immunization acts like an infection, initiating an innate immune response and then activating an antigen-specific adaptive immune response. Each pathogen has its mechanism and can induce different humoral or cell-mediated adaptive immune responses (72). Specifically, humoral immunity, which depends on antibody effector mechanisms such as neutralization, opsonization, and complement activation, appears to be the main mechanism involved in the elimination of extracellular bacteria (73). In addition to traditional immunization methods, passive immunization involves the administration of pre-formed antibodies, providing immediate protection against infections. This approach can offer rapid immunity, particularly in acute infections and when dealing with resistant pathogens like *A. baumannii* (74, 75).

Passive immunization acts as a form of “borrowed” immunity against a disease or a pathogen, serving as a treatment for patients with already installed infections. However, it provides only temporary immunity since the host does not develop an immune response as in active immunization. This treatment lasts only as long as the transferred antibodies persist, without inducing immunological memory. Nevertheless, it may be sufficient to clear an acute infection, either alone or in combination with antimicrobials that have less than optimal efficacy (74).

Since 1890, passive immunization has been used in humans, starting with immune sera from animals to treat infected children (76). Most recently, several infectious diseases have been addressed with human immune globulin. This treatment has been effective for tetanus and rabies for over 50 years, treating non-immunized patients who have had potential exposure to the pathogen. Additionally, monoclonal antibody strategies have also been developed for diseases like varicella, cytomegalovirus, hepatitis B, botulism, and respiratory syncytial virus (77). Broadly neutralizing antibodies have also shown promise in animal models for HIV treatment, yielding significant results (78). These specific antibodies possess high specificity and affinity, contributing to their potent efficacy. Their functional activities include agglutination, mucus trapping, inhibition of attachment and fusion to target cells, neutralization, complement activation, and phagocytosis via Fc receptors (76).

Understanding how immunotherapies stimulate the immune system and their potential side effects is crucial for predicting their effectiveness and specificity. Seven studies took a distinct approach, employing polyclonal or monoclonal antibodies against recombinant adhesins to understand their protective mechanisms against *A. baumannii in vitro* (23, 26, 27, 33–36).

Although there are no commercially available products for polyclonal antibody preparations, clinical trial data already show promise for further research in this area. Polyclonal antibodies are generally more cost-effective and easier to prepare compared to mAbs, making them an attractive option for initial studies (76). Four of these studies adopted the approach of investigating the antibiofilm activity of antibodies against recombinant proteins. In these studies, mice were immunized with recombinant proteins, and the resulting serum polyclonal antibodies were employed against biofilm formation (23, 26, 35, 36). Disrupting biofilm formation represents a significative alternative for treating *A. baumannii* and is also an interesting strategy for controlling abiotic biofilm formations in healthcare institutions. Biofilms are an important virulence factor, allowing this species to grow and survive in harsh conditions (6, 79).

The anti-sera targeting Ata protein demonstrated the ability to disrupt *A. baumannii* biofilm formation across different culture broths. This finding is notably significant as it is well established that different species exhibit varying adherent capacities influenced by numerous factors, such as culture media (6). Additionally, serum anti-rCAM87009.1 and anti-rFilF could inhibit biofilm formation in clinical isolates exhibiting carbapenem-resistant *A. baumannii* strains (35, 36). These strains have been considered the top priority on the World Health Organization’s list for antimicrobial development because they constitute 20% of infections in hospitalized intensive care unit (ICU) patients and 7% of infections in patients with medical devices like polystyrene catheters and titanium prosthetics. In such instances, biofilm formation emerges as a major virulence factor, facilitating colonization on both biotic and abiotic surfaces, including medical devices (80). Barati *et al*. (26) investigate the role of outer membrane protein A (OmpA) in *A. baumannii* biofilm formation and adherence. They demonstrate that anti-OmpA antibodies effectively inhibit biofilm formation, as well as the adherence and proliferation of *A. baumannii* within epithelial cells. Additionally, OmpA-induced antibodies demonstrated potent bactericidal effects (26).

The trimeric autotransporter adhesin family member, Ata, is known as a key virulence factor in *A. baumannii* and has been identified multiple times as a potential vaccine target. To understand how this antigen modulates the immune system, Sun *et al*. (34) evaluated the opsonic activity of specific antibodies generated against Ata. They incubated HL60 cells with *A. baumannii* ATCC 17978, human complement, and heat-treated anti-rAta antibodies to measure the ability of these antibodies to opsonize *A. baumannii* cells and induce phagocytic killing by HL60 cells in the presence of human complement. Compared with the naïve serum group, bacteria killed by HL60 cells increased to approximately 63%, indicating that one method of action of Ata protein immunization is the induction of opsonization followed by phagocytosis (34). After discovering that passive immunization with anti-rNucAb antibodies in mice challenged with *A. baumannii* infection resulted in a doubled survival rate compared to mice actively immunized with rNucAb, Garg *et al*. (27) conducted a similar complement-mediated serum bactericidal assay. However, no enhancement in bacterial killing was observed. This methodology excluded complement-mediated killing as the mechanism of action of anti-rNucAb antibodies. The authors suggest that the bacteria retained their serum resistance capability against complement (27).

NucAb epitope vaccination was further assessed for its impact on host adhesive capability. It is well established that collagen type V represents a component of the host extracellular matrix, with specific structural features that may enhance bacterial adhesion by serving as a binding substrate for bacteria. Ren and colleagues (33) constructed a T- and B-cell multi-epitope (rMEP) chimera comprising outer membrane proteins NucAb and FilF, which are known for their promising role in bacterial adhesion to host cells and surfaces. The rMEP, as previously mentioned, was evaluated for its immunoprotective response in mice challenged with *A. baumannii*, but the research also extended to analyze the anti-adhesive effects of this vaccination. Mouse antibodies against rMEP were evaluated for their capacity to inhibit bacterial adherence to collagen type IV. Incubating the standard strain *A. baumannii* ATCC 19606 with anti-rMEP antibodies resulted in decreased bacterial cell binding (33). This multifaceted exploration underscores the diverse applications and methodologies within the theme of recombinant DNA techniques in the surveyed literature.

### Further insights into the role of adhesins in *A. baumannii* infections

Abirami *et al*. (30) pursued another approach by highlighting pyrogallol, an anti-infective, anti-biofilm, and anti-virulence compound, for its potential to suppress *A. baumannii* virulence. They observed that 12 proteins involved in virulence mechanisms were downregulated in *A. baumannii* cells treated with pyrogallol. Among these, seven proteins (ClpB, Bap, Csu A/B, PgaA, PgaC, BfmR, and OmpA) were implicated in bacterial adhesion, biofilm formation, and invasion of host cells (30). This group of proteins represents key features of *A. baumannii*’s virulence mechanisms, thereby highlighting potential targets for controlling infections. ClpB is one of the heat shock proteins that play a vital role in bacterial survival under hostile conditions by preventing cell damage and is also linked to antibiotic resistance. This mechanism enhances *A. baumannii*’s resistance to β-lactam antibiotics (81). Additionally, Bap, CsuA/B, OmpA, and BfmR are adhesin proteins that contribute to surface colonization and biofilm formation, while PgaA and PgaC are linked to the biosynthesis of poly-β−1,6-N-acetylglucosamine, a major component of the extracellular matrix in biofilms (82). This observation suggests that therapies against *A. baumannii* should target adhesin proteins, which play a crucial role in the bacteria’s virulence mechanisms.

## CONCLUDING REMARKS ON ADHESIN TARGETING AS A STRATEGY FOR NEW THERAPEUTICS AGAINST *A. baumannii* INFECTIONS

In this review, we present over 20 adhesin targets with significant potential for controlling *A. baumannii* infections. Various study strategies were highlighted, including *in vivo*, *in vitro*, and *in silico* approaches. Most of the adhesins included in this systematic review play a fundamental role in biofilm formation, which is one of the main virulence factors of this pathogen, enabling the colonization of both biotic and abiotic surfaces, particularly in hospital environments. By tracking and summarizing targets for infection control and highlighting their role in the pathogenesis of *A. baumannii*, we aim to aid in the selection of epitopes for further exploration in therapies and prevention of these infections.
